# *In vitro* Digestion Characteristics of Hydrolyzed Infant Formula and Its Effects on the Growth and Development in Mice

**DOI:** 10.3389/fnut.2022.912207

**Published:** 2022-06-24

**Authors:** Lifang Feng, Wei Ye, Kuo Zhang, Daofeng Qu, Weilin Liu, Min Wu, Jianzhong Han

**Affiliations:** ^1^School of Food Science and Biotechnology, Zhejiang Gongshang University, Hangzhou, China; ^2^Ecology and Health Institute, Hangzhou Vocational and Technical College, Hangzhou, China

**Keywords:** hydrolyzed infant formula, gastrointestinal digestion model, mouse, growth and development, fecal microbiota

## Abstract

Infant formula, an important food for babies, is convenient and nutritious, and hydrolyzed formulas have attracted much attention due to their non-allergicity. However, it is uncertain whether hydrolyzed formulars cause obesity and other side effects in infants. Herein, three infant formulas, standard (sIF), partially hydrolyzed (pHIF), and extensively hydrolyzed (eHIF), were analyzed in an *in vitro* gastrointestinal digestion model. With increasing degree of hydrolysis, the protein moleculars, and allergenicity of the proteins decreased and the long-chain polyunsaturated fatty acid content increased. Moreover, the digestion model solutions quickly digested the small fat globules and proteins in the hydrolyzed formula, allowing it to become electrostatically stable sooner. The eHIF-fed mice presented larger body sizes, and exhibited excellent exploratory and spatial memory abilities in the maze test. Based on villus height and crypt depth histological characterizations and amplicon sequencing, eHIF promoted mouse small intestine development and changed the gut microbiota composition, eventually favoring weight gain. The mouse spleen index showed that long-term infant formula consumption might be detrimental to immune system development, and the weight-bearing swimming test showed that eHIF could cause severe physical strength decline. Therefore, long-term consumption of infant formula, especially eHIF, may have both positive and negative effects on mouse growth and development, and our results might shed light on feeding formula to infants.

## Introduction

Breast milk is nutritionally balanced and rich in immune components and growth factors. Breast milk plays an important and even irreplaceable role during the growth and development of infants and in improvement of their immune system (1). However, due to factors such as the modern fast-paced lifestyle, high-intensity work and stress, some women encounter problems with an insufficient supply or lack of milk, so infant formula is provided as a staple food to infants. A 2015 survey of 459 mother-infant pairs in seven Chinese cities indicated that from 0∼3 to 0∼6 months, the proportions of pairs exclusively breastfeeding were 34.4 and 14.1%, respectively, and the proportions of pairs predominantly breastfeeding were 61.6 and 55.6%, respectively (2). Another survey conducted by the National Immunization Survey of U.S. from 2011 to 2018 reported that the proportions of infants exclusively breastfeeding at 0∼3 and 0∼6 months were about 45 and 24%, respectively (3). Therefore, infant formula has a very broad market.

Cows naturally produce a high yield of milk, which is rich in protein, so cow’s milk is the main raw material for infant formula. However, some differences remain between cow’s milk and breast milk. For example, the ratio of whey to casein (CN) in mature breast milk is 60:40, while this value is 20:80 in cow’s milk (4, 5). In addition, β-lactoglobulin (β-LG) accounts for approximately 50% of the total whey protein in cow’s milk, but this protein is completely absent from the whey of breast milk; moreover, β-LG is thought to be the major allergen in cow’s milk, with the disulfide bonds being responsible for the allergic reactions (6). Cow’s milk is nutrient-rich, but compared to breast milk, it lacks certain nutrients; for example, long-chain polyunsaturated fatty acids (LCPUFAs) composed of 20–22 carbon atoms are essential for retina and nervous system development and function, but n-3 and n-6 LCPUFAs are present in only breast milk (7). Compared to cow’s milk, the LCPUFAs in breast milk contain more high-affinity sn-2 and sn-3 sites that are highly compatible with lipases, leading to the hydrolysis of long-chain fatty acids (8). Bile salt-stimulated lipase, an important lipase involved in duodenum and lipid digestion, enables breast-fed infants to digest lipids in the small intestine more easily (9, 10). The lactoferrin in breast milk has an immunological function as an iron transporter that stimulates infant mucosa proliferation and enhances antibacterial effects (11). Considering the differences between cow’s milk and breast milk, the composition of cow’s milk has been adjusted to make infant formulas that more closely resemble breast milk by adding or removing various supplements (4).

In addition, since some newborns are severely allergic to cow’s milk, the macromolecular proteins in cow’s milk are degraded into short peptides and amino acids through hydrolysis technology to eliminate allergenic proteins (12, 13). Currently, infant formulas on the market include partially hydrolyzed infant formula (pHIF) and extensively hydrolyzed infant formula (eHIF); the former can be used to prevent cow’s milk protein allergy, and the latter can be given to infants with cow’s milk allergy (1). Some consumers prefer to choose pHIF or eHIF over standard infant formula (sIF) to avoid potential allergic issues. Niggemann et al. found that the body lengths and head circumferences of infants fed eHIF were not affected, but these infants weighted slightly less than their counterparts fed sIF (14). Blake-Lamb et al. conducted 34 survey reports, of which 9 valid results showed an increased risk of childhood obesity in infants fed hydrolyzed infant formula (15). However, Knip et al. reported that the use of hydrolyzed infant formula did not affect the cumulative incidence of type 1 diabetes after 11.5 years of follow-up in 2,159 infants (16).

Controversial research reports on the effects of hydrolyzed infant formula on infant health suggest that more experimental evidence is needed to elucidate the digestive trajectories of hydrolyzed infant formula and its effects on the growth and development of infants or experimental mammals after feeding. The advantages and disadvantages of hydrolyzed infant formula need to be clarified to provide guidance to both formula manufacturers and home consumers. In this study, infant formulas with different degrees of hydrolysis were digested in an *in vitro* gastrointestinal digestion model, and their digestion trajectories and products were analyzed. In addition, mice were fed sIF, pHIF, and eHIF and their body size, intelligence, physical strength, organ indexes, and fecal microbiota were studied to determine the effects of hydrolyzed infant formula on mouse growth and development.

## Materials and Methods

### Materials

Pepsin (10 FIP-U/mg), trypsin (4 FIP-U/mg), bile salt, imidazole, Fast green, Nile red, and standard mixed fatty acids were purchased from Sigma–Aldrich (Darmstadt, Germany), while the remaining chemical reagents were purchased from Sinopharm (Shanghai, China). Three different brands of infant formula were purchased from a local supermarket. To avoid potential conflicts of commercial interest, they were named A, B, and C. Each brand contained three infant formulas with different degrees of hydrolysis (sIF, pHIF, and eHIF). The nutrients in these formulas are listed in Supplementary Table 1.

### The *in vitro* Gastrointestinal Digestion Model

Powdered sIF, pHIF, and eHIF were reconstituted with Milli-Q water according to the product label instructions. Based on an *in vitro* infant digestion model (17) and INFOGEST (18), an *in vitro* gastrointestinal digestion model was constructed to mimic the infant digestion trajectory. The simulated gastric fluid (SGF) was composed of 0.15 mM CaCl_2_, 150 mM NaCl, and 7 M HCl at pH 3.0, and the simulated intestinal fluid (SIF) was composed of 0.6 mM CaCl_2_, 150 mM NaCl, 6.8 g/L K_2_HPO_4_, and 0.2 M NaOH at pH 7.0.

The process of gastric digestion was as follows. First, 28 mL of reconstituted formula was mixed with 14 mL of SGF, and the pH was adjusted to 4.0 with 1 M HCl. Subsequently, 268 U/mL pepsin and 19 U/mL gastric lipase were added, and the digestion process was performed in a 37°C water bath with shaking at 140 rpm.

The process of intestinal digestion was as follows. First, 20 mL of gastric digestive products was mixed with 20 mL of SIF, and the pH was adjusted to 6.6 with 0.1 M NaHCO_3_. Then, 90 U/mL trypsin and 3 mM bile salt were added, and the digestion process was carried out according to the same procedure used for gastric digestion.

### Detection of Protein Components During *in vitro* Digestion

One milliliter of digestive product was inactivated by boiling for 0, 30, 60, 90, 120, 150, and 180 min. The fat in the supernatant was discarded after centrifugation at 1,000 rpm for 10 min, and then the intermediate layer of liquid was transferred to a new tube and stored at 4°C. All samples were loaded into the wells of an SDS–PAGE gel (Nanjing Jiancheng Bioengineering Institute, Nanjing, China), which was run at 30 V until the dye front migrated into the running gel (∼10 min), and increased to 100 V until the dye front reached the bottom of the gel (∼1 h). Gels were fixed in the solution (0.5% glutaraldehyde and 30% ethanol) for 30 min, and then stained in the Coomassie solution for 1.5 h on a shaker. Finally, gels were rinsed in the destaining solution (10% isopropanol and 10% glacial acetic acid) for 2 h (19).

### Detection of Allergenic Proteins During *in vitro* Digestion

Half a milliliter of the digestive product was mixed with 10 mL of dilution buffer from a milk (casein and β-lactoglobulin) ELISA kit (catalog number: EKT-ML60, Pribolab, Qingdao, China), and then incubated in a 60°C water bath for 15 min. After centrifugation at 2,000 rpm for 10 min, the allergenic proteins in the supernatant and pellet were detected by the same ELISA kit according to the manufacturer’s protocol.

### Detection of Lipid Components During *in vitro* Digestion

Two milliliters of reconstituted formula was mixed with 26.67 mL of chloroform and 13.33 mL of methanol, and the mixture was sonicated for 20 min. After centrifugation at 3,500 rpm for 5 min, the organic phase was transferred to a rotary evaporator for evaporation of the solvent, and the residue was dissolved in 1 mL of n-hexane. Then, 30 mL of the extract solution was mixed with 0.7 mL of 10 M KOH solution and 5.3 mL of methanol for 90 min of incubation in a 55°C water bath. After that, 0.58 mL of 12 M H_2_SO_4_ was added, and the solution was incubated for an additional 90 min. Next, 3 mL of n-hexane was added, and the solution was vortexed for 5 min followed by centrifugation at 3,500 rpm for 10 min. The supernatant was transferred to a glass tube containing 1 mM anhydrous sodium sulfate. After 10 min, the solution was transferred to a gas chromatography (GC) vial and stored at −18°C.

Lipid composition was determined by gas chromatography–mass spectrometry (GC–MS, 7890/5975, Agilent, Santa Clara, CA, United States). Compounds were separated on a DB-WAX column (60 m × 0.25 mm × 0.5 μm, Agilent, Santa Clara, CA, United States). The column temperature was held at 50°C for 1 min, then increased to 200°C at a rate of 20°C/min, then further increased to 240°C at a rate of 3°C/min, and maintained 25 min. All data were collected with a mass scan range of 33–500 amu (20).

### Microstructural Characterization and Zeta Potentials of the Proteins and Lipids During *in vitro* Digestion

Digestion products were stained with Nile red and Fast green and incubated in the dark for 30 min. The samples were photographed with a confocal microscope (LSM710, Jena, Germany). Intestinal digestive products were diluted 100-fold with citrate (pH 1.2) and PBS, and their zeta potential values were measured with a Zetasizer Nano (Malvern, Malvern, Worcestershire, United Kingdom).

### Evaluation of the Growth and Intelligence of Mice Fed Infant Formula

The *in vivo* effects of brand B infant formula with different degrees of hydrolysis were evaluated in mice by measuring their growth and development and utilizing a maze test and a weight-bearing swimming test. Forty ICR mice of 3–4 weeks old were randomly divided into 4 groups (10 mice per group), and each cage contained one male mouse. Three groups were fed infant formula (sIF, pHIF, or eHIF), while the control group was fed normal chow. During the 30-day feeding period, the body weights, body lengths, and tail lengths of the mice were measured every 5 days.

For the maze test, the Y-shaped maze consisted of three arms. The angle between each arm was 120°, and the size of each arm was 50 cm × 18 cm × 30 cm. Each arm had a movable partition board at the intersection, and the interior of the maze was dark. A mouse was placed at the end of any arm and was free to explore for 8 min. The following indexes were recorded: (a) total number of arm entries; one point was recorded if a mouse stepped into any arm; and (b) number of turns; a turn was recorded if a mouse entered three arms (21).

For the weight-bearing swimming test, a lead block with a mass 5% that of the mouse weight was attached to the mouse’s tail. The mice were placed in the swimming tank, and their swimming time was recorded starting when the mouse began to swim until the whole body below the water surface (22).

After the above experiments, mice were euthanized by ether inhalation. Samples of liver, kidney, and spleen were collected aseptically and weighed. The organ index was calculated using the following formula (23):


$$O\,r\,g\,a\,n\,i\,n\,d\,e\,x=\frac{\mathrm{Organ}\,\mathrm{weight}}{\mathrm{body}\,\mathrm{weight}}\times 100$$

In addition, ileal samples were cut and fixed in 4% paraformaldehyde. The samples were then dehydrated with a gradient of ethanol solutions (75% for 4 h, 85% for 2 h, 90% for 2 h, 95% for 1 h, and 100% for 1 h). Subsequently, the samples were transferred to a solution of xylene and ethanol (v:v = 1:1) for 30 min and immersed in xylene until transparent. After treatment with toluene for 30 min, the samples were placed in a warm paraffin wax bath for 1 h, and this step was repeated three times. Next, the samples were placed in a block of wax and cut into slices with a thickness of 4 μm. Slices were dewaxed twice with xylene for 20 min, deparaffinized with xylene and ethanol (v:v = 1:1) for 10 min, and then treated with a gradient of ethanol solutions (100, 95, 90, 80, and 70%) for 5 min at each concentration. After 10 min of hematoxylin staining, samples were dehydrated with a gradient of ethanol solutions (70, 80, and 90%) for 2 min at each concentration. Slices were stained with eosin for 2 min and subsequently treated with 90% ethanol solution, 100% ethanol solution and xylene for 2 min each. Finally, the slices were sealed with gum, and the specimens were photographed (24). All experiments were approved by the School Animal Care and Ethics Committee of Zhejiang Gongshang University.

### Analysis of Fecal Microbiota

On day 30, 3 mice were randomly selected from each group (before maze test and weight-bearing swimming test), and their stool samples were collected, and fecal genomic DNA was extracted using the TIANamp Stool DNA Kit (Tiangen, Beijing, China). The V3+V4 region of the 16S rRNA gene was amplified by primers of 16S_f: 5′-ACTCCTACGGGAGGCAGCAG-3′ and 16S_r: 5′-GGACTACHVGGGTWTCTAAT-3′, and the PCR products were purified with an AxyPrep PCR Cleanup Kit (Axygen, Union City, CA, United States). After qualification by real-time quantitative PCR (Applied Biosystem, Carlsbad, CA, United States), purified PCR products were sequenced by the Illumina MiSeq System (2 × 300 bp). All Illumina DNA sequencing data were submitted to the NCBI Short Read Archive with accession number PRJNA822855. High-quality sequences were extracted, and unique sequences were subjected to the Ribosomal Database Project database (version 10.28) (25). The 16S rRNA gene amplicon sequences were identified into amplicon sequence variants (ASVs) using Qiime2 software and DADA2 pipeline. The Shannon diversity index was used to assess the bacterial alpha-diversity, and principal component analysis (PCA) and principal coordinate analysis (PCoA) were performed to reveal the fecal microbiota produced by feeding infant formulas with different degrees of hydrolysis.

### Statistical Analyses

All analyses were carried out in triplicate, and results are expressed as mean ± standard deviation (*SD*) of triplicate determinations. One-way analysis of variance (ANOVA) was performed on all data, and the mean separation was calculated by Tukey’s multiple range test using SPSS software.

## Results and Discussion

### Infant Formulas With Different Degrees of Hydrolysis Were Composed of Different Sized Proteins

The proteins in cow’s milk can be degraded by both heat and enzymatic digestion, and these technologies reduce the allergenicity of milk proteins and the probability of cow’s milk protein allergy (CMPA) in infants fed hydrolyzed infant formula. A meta-analysis found that infants fed hydrolyzed infant formula had a lower risk of atopic dermatitis than those fed sIF (26). To determine the digestion trajectories of standard and hydrolyzed infant formula, we used an *in vitro* digestion model, which has the advantages of low cost, a lack of ethical restrictions, and reduced time requirements (1).

**FIGURE 1:**
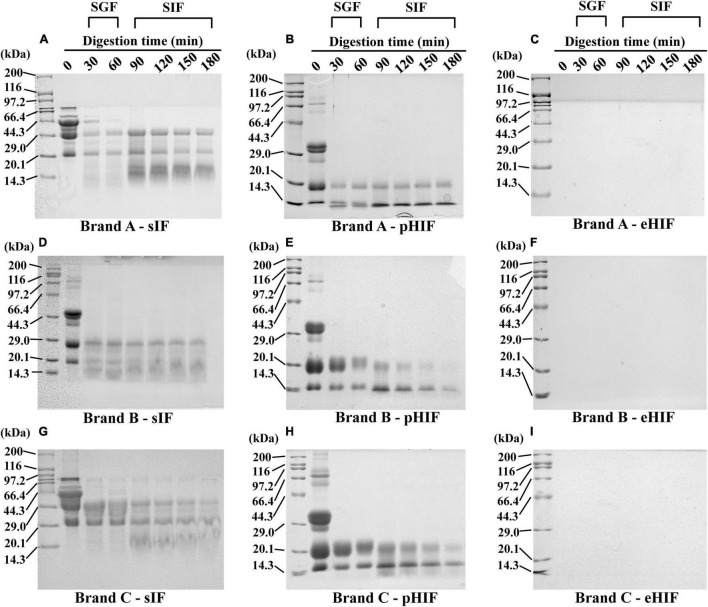
SDS–PAGE. **(A–C)** Represent protein electrophoresis of the brand A of the sIF, pHIF, and eHIF after SGF and SIF digestion, respectively. **(D–F)** Represent protein electrophoresis of the brand B of the sIF, pHIF, and eHIF after SGF and SIF digestion, respectively. **(G–I)** Represent the protein electrophoresis of the brand C of the sIF, pHIF, and eHIF after SGF and SIF digestion, respectively.

SDS–PAGE is a convenient and effective method for protein analysis that can indicate the composition of proteins in a sample and the formation of peptides larger than 3.5 kDa (19). The proteins from sIF ranged in size from 20.1 to 116 kDa, with many being approximately 44 kDa. During heating, the free thiol group of β-LG (18 kDa) binds to CN (∼25 kDa) to form a disulfide cross-linked complex (β-LG-CN). β-LG also binds α-lactalbumin (α-La, 14 kDa) by either disulfide cross-linking or hydrophobic interactions to form aggregates (β-LG-α-La) (27). After 30 min of SGF digestion, many proteins > 29 kDa were hydrolyzed, and hydrolysis was almost complete after 60 min. During SIF digestion, proteins > 29 kDa were completely hydrolyzed to small proteins 14.3∼20.1 kDa in size (Figures 1A,D,G). This result suggested that proteins with larger molecular weights, such as the β-LG-CN and β-LG-α-La complexes, bovine serum albumin (66.5 kDa), and lactoferrin (78 kDa) (28, 29), had been digested, and our *in vitro* digestion model worked well.

Most of the proteins from pHIF ranged from 29 to 44.3 kDa, and almost no proteins were larger than 66.4 kDa, indicating that the protein composition of this type of hydrolyzed infant formula differed from that of sIF, as few proteins had large molecular weights. After 30 min of SGF digestion, proteins in the range of 29∼44.3 kDa were completely degraded, and those weighing 14.3∼20.1 kDa also began to gradually degrade. During SIF digestion, proteins weighing 20.1 kDa were degraded, especially after 180 min. In addition, the 14.3 kDa proteins from brand A remained unchanged during the SIF digestion process. However, proteins of the same size in brands B and C had been degraded (Figures 1B,E,H), suggesting that the protein composition of infant formula varied from company to company.

No protein bands were detected from the three brands of eHIF (Figures 1C,F,I), indicating that the components of eHIF were almost all peptides. This result was consistent with previous reports that hydrolyzed infant formula was mainly composed of digestible small-molecular-weight proteins and peptides rather than large-molecular-weight proteins (30). These data raised several questions. What made hydrolyzed infant formula easy to digest? Additionally, what were the effects of hydrolyzed infant formula on mammalian growth and development? Before these questions could be answered, we first needed to understand the characteristics of hydrolyzed infant formula.

### The Allergenic Protein Content in Infant Formula Decreased During *in vitro* Digestion

**FIGURE 2:**
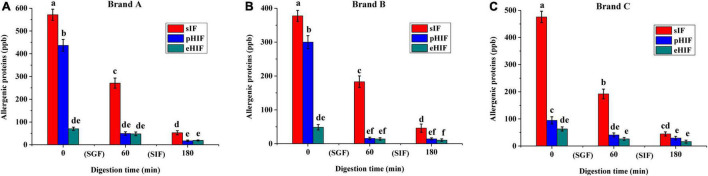
The contents of allergenic proteins in infant formula, *n* = 3 independent experiments. **(A–C)** Represent infant formula of brands (a–c) during SGF and SIF digestion stages, respectively.

Both β-LG and CN are major allergens leading to CMPA in infants (31), so their contents can indicate the extent of the allergenic proteins in infant formula. In the undigested stage, pHIFs from brands A and B contained a large amount of allergenic proteins (Figures 2A,B), but brand C contained almost no allergenic proteins (Figure 2C), which further confirmed that the infant formula protein composition varied from company to company. After SGF digestion, the contents of allergenic proteins in the three brands of sIF were reduced by approximately half, while those in pHIF were completely digested. After SIF digestion, allergenic proteins in all sIF samples were fully digested. These results showed that allergenic proteins present in pHIF could be easily digested and were almost absent in eHIF, which was an important reason why the hydrolyzed infant formula did not cause CMPA in infants (32).

### The Absolute Zeta Potentials of Protein and Lipid Surfaces in Infant Formula Increased During *in vitro* Digestion

Changes in the surface charges of CN and fat globules can promote digestive speed, so zeta potential measurements are suitable to characterize the surface charge changes in these particles and the digestion efficiency of milk (33). When the absolute value of the zeta potential is greater than 30 mV, the digestive system has good electrostatic stability (34). The zeta potentials of the three brands of infant formula showed similar trends during digestion (Table 1). After 45 min of SGF digestion, the zeta potentials of all samples did not change substantially and were basically stable at approximately 2 mV, which indicated that the electrostatic stability of the infant formula in the digestive solution was poor. The zeta potential of cow’s milk was evidently affected by varying pH (35). After 60 min of SGF digestion, at which point no additional manual pH adjustment was made, the zeta potentials of sIF and pHIF increased to approximately 10 mV, while that of the eHIF sample dropped sharply to −40 ∼−50 mV, indicating that as the infant formula was digested, the contact area between fat globules and enzymes increased, the interfacial reaction was accelerated, and the digested eHIF solution reached an electrostatically stable state more quickly.

**TABLE 1:** Changes of zeta potential (mV) in infant formula during *in vitro* digestion.

Digestion time (min)	Brand A			Brand B			Brand C		
			
	sIF	pHIF	eHIF	sIF	pHIF	eHIF	sIF	pHIF	eHIF
0	1.49 ± 0.19*^b^*	0.94 ± 0.12*^b^*	0.02 ± 0.37*^a^*	1.66 ± 0.20*^b^*	1.23 ± 0.13*^a^*	−0.10 ± 0.17*^a^*	2.30 ± 0.24*^b^*	1.35 ± 0.06*^b^*	−0.14 ± 0.02*^a^*
15	1.89 ± 0.45*^b^*	0.87 ± 0.02*^b^*	0.09 ± 0.04*^a^*	1.59 ± 0.44*^b^*	1.41 ± 0.04*^a^*	0.10 ± 0.10*^a^*	2.37 ± 0.07*^b^*	1.34 ± 0.19*^b^*	−0.24 ± 0.13*^a^*
30	1.94 ± 0.26*^b^*	1.32±0.23ba	0.96 ± 0.14*^a^*	1.75 ± 0.12*^b^*	1.25 ± 0.16*^a^*	0.91 ± 0.16*^a^*	2.66 ± 0.75*^b^*	1.80 ± 0.23*^b^*	−0.17 ± 0.10*^a^*
45	1.76 ± 0.20*^b^*	1.35±0.23ba	1.27 ± 0.15*^a^*	1.58 ± 0.22*^b^*	1.16 ± 0.09*^a^*	0.62 ± 0.09*^a^*	2.48 ± 0.32*^b^*	1.61 ± 0.08*^b^*	−0.12 ± 0.23*^a^*
60	12.27 ± 0.32*^a^*	6.23 ± 1.04*^a^*	−48.30 ± 0.20*^b^*	7.94 ± 1.66*^a^*	4.13 ± 0.47*^a^*	−42.90 ± 1.68*^b^*	11.07 ± 1.50*^a^*	9.80 ± 0.59*^a^*	−47.47 ± 3.25*^b^*
90	−30.87 ± 1.31*^c^*	−40.63 ± 2.72*^c^*	−51.23 ± 4.11*^b^*	−41.33 ± 1.80*^c^*	−38.33 ± 0.61*^b^*	−53.50 ± 1.05*^c^*	−25.70 ± 0.62*^c^*	−42.47 ± 3.66*^c^*	−48.43 ± 2.12*^b^*
120	−31.23 ± 1.37*^c^*	−37.90 ± 2.79*^c^*	−51.00 ± 1.81*^b^*	−41.70 ± 2.34*^c^*	−41.10 ± 2.61*^b^*	−50.73 ± 3.26*^c^*	−26.77 ± 1.43*^c^*	−42.23 ± 1.29*^c^*	−49.97 ± 3.16*^b^*
150	−30.67 ± 2.05*^c^*	−39.17 ± 3.33*^c^*	−51.27 ± 2.10*^b^*	−41.63 ± 0.72*^c^*	−38.90 ± 1.39*^b^*	−50.83 ± 3.10*^c^*	−28.07 ± 1.01*^c^*	−42.27 ± 1.33*^c^*	−48.87 ± 1.46*^b^*
180	−32.00 ± 1.21*^c^*	−38.67 ± 1.48*^c^*	−52.53 ± 1.10*^b^*	−42.13 ± 2.44*^c^*	−43.03 ± 1.11*^b^*	−51.77 ± 1.59*^c^*	−27.23 ± 1.53*^c^*	−42.40 ± 2.20*^c^*	−47.70 ± 2.67*^b^*

*Means in same column with different small letters are significantly different (p < 0.05).*

Under the action of bile salt and pancreatin, the abundant negative charges on the surface of bile salt can promote the hydrolysis of fat globules and produce monoglycerides, diacylglycerol, free fatty acids, and other substances, which accumulated on the interface of fat globules (36). During SIF digestion, the zeta potentials of sIF and pHIF decreased rapidly, and the reaction systems of these two samples reached an electrostatically stable state, which might be due to the interaction between the digested proteins and lipid products with the bile salt phospholipid micelles and phospholipids in vesicles.

### Characteristics of Fatty Acids in Infant Formula During *in vitro* Digestion

Fatty acids are one of the most important components in infant formula; approximately 85% are saturated and monounsaturated fatty acids, and the rest are polyunsaturated fatty acids. These fatty acids are an important source of energy for cells, a principal substance constituting cell membrane components, and precursors of many metabolic compounds (e.g., prostacyclin, prostaglandin, thromboxane, and leukotriene) (37). Ten fatty acids were detected in this study, and the contents of c12:0, c16:0, c18:1, and c18:2 exceeding 15 g/100 g in the undigested (control) and all digested (SGF and SIF) samples. Mendonça et al. determined the contents and profile of fatty acids in 10 standard infant formulas and found that only c16:0 exceeded 15 g/100 g (20), suggesting that the fatty acid content of different brands of infant formula varied widely.

**FIGURE 3:**
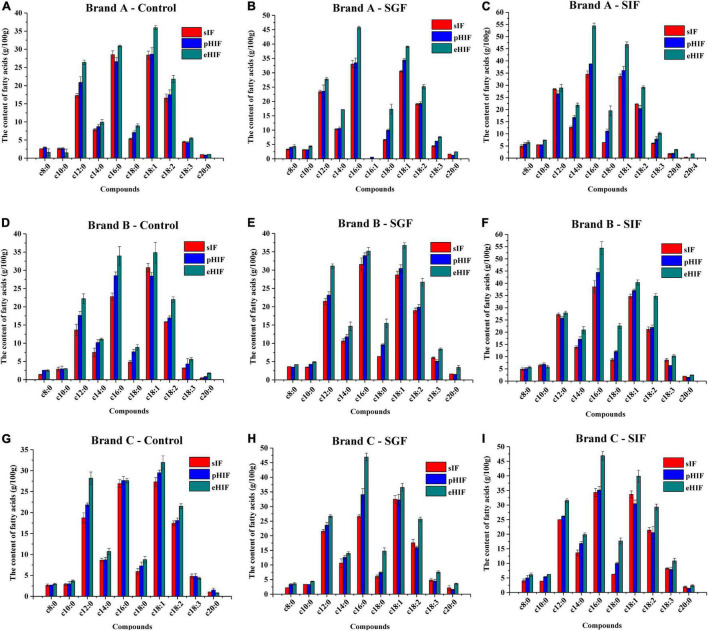
Characteristics of fatty acids in infant formulas during *in vitro* digestion, *n* = 3 independent experiments. **(A–C)** Represent the brand A infant formulas during control, SGF, and SIF digestion stages, respectively. **(D–F)** Represent the brand B infant formulas during control, SGF, and SIF digestion stages, respectively. **(G–I)** Represent the brand C infant formulas during control, SGF, and SIF digestion stages, respectively.

Before digestion, the contents of c12:0, c14:0, c16:0, c18:0, c18:1, c18:2, and c18:3 were higher in eHIF than in pHIF and sIF (Figures 3A,D,G). During SGF digestion, gastric lipase preferentially broke down sn-3 fatty acids into diglycerides containing sn-1, sn-2, and free fatty acids, resulting in more LCPUFA production in eHIF than in sIF and pHIF (Figures 3B,E,H). During SIF digestion, the fatty acid contents in eHIF were also higher than that in sIF and pHIF, especially LCPUFAs (Figures 3C,F,I). Numerous studies have shown that LCPUFAs containing n-3 and n-6 fatty acids (e.g., α-linoleic acid C18:3, n-3; linoleic acid C18:2, n-6) contribute to the infant nervous system and retina development (38, 39), suggesting that eHIF had a better nutrient composition and promoted brain development after digestion. However, the contents of most fatty acids were not significantly different between sIF and pHIF samples.

The SDS–PAGE results displayed that brand B was similar to brand C. Additionally, the allergenic protein detection results showed that brand B was similar to brand A. Finally, with respect to the zeta potential and fatty acid analyses, the three brands were similar. Thus, to eliminate the unnecessary duplication of experiments, only brand B was used in the following experiments.

### Proteins and Lipids in Infant Formula Depolymerized During *in vitro* Digestion

**FIGURE 4:**
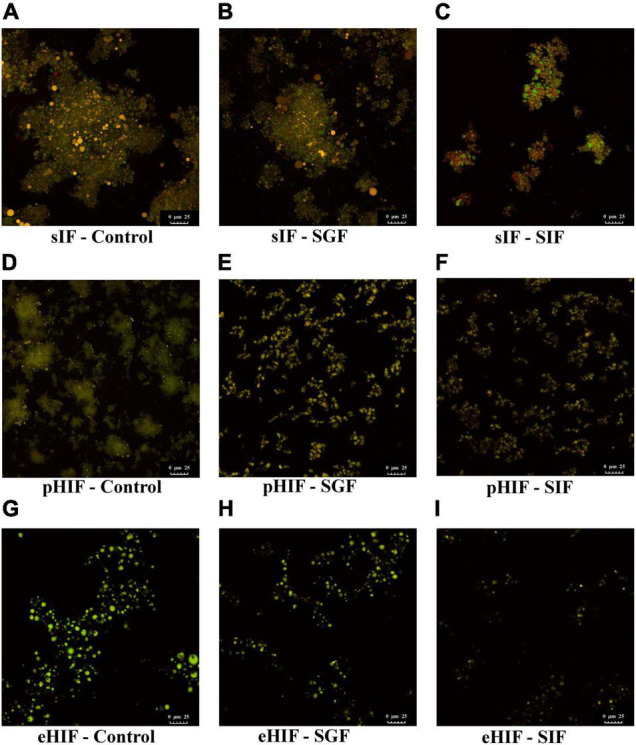
Microstructures of proteins and lipids in infant formulas during *in vitro* digestion. **(A–C)** Represent the sIF during control, SGF, and SIF digestion stages, respectively. **(D–F)** Represent the pHIF during control, SGF, and SIF digestion stages, respectively. **(G–I)** Represent the eHIF during control, SGF, and SIF digestion stages, respectively.

Fluorescent staining is an important method for determining the microstructures of proteins and lipids. When bound to proteins or lipids, Fast Green and Nile Red can emit fluorescence at 633 and 488 nm, respectively (40). Before digestion, both pHIF and eHIF had a higher number of smaller proteins and fat globules (Figures 4D,G) than sIF (Figure 4A). Several studies have reported that fat globule size is associated with formula digestibility; colostrum and mature breast milk have larger fat globules (9 and 4 μm, respectively) and are digested more slowly than infant formula (0.4 μm) (41, 42). Therefore, smaller fat globules might allow for faster digestion of hydrolyzed infant formula in subsequent experiments.

Patel et al. proposed that gastric lipase digest lipids in the infant stomach, encouraging pancreatic lipase activity by creating a better interface between the aqueous environment and fat globules (43). During SGF digestion, for sIF, fat globule size was further reduced, lipid droplets were slightly precipitated by gastric lipase, and proteins were digested by pepsin (Figure 4B). For pHIF, fat globules were smaller in size than those from sIF, and lipid droplets did not precipitate and were surrounded by flocculent proteins (Figure 4E). For eHIF, almost no protein was detected, and only a few fat globules remained (Figure 4H).

During SIF digestion, for sIF, many proteins and lipids remained undigested, and lipid droplets showed obvious precipitation (Figure 4C). For pHIF, small amounts of lipids were encapsulated by proteins (Figure 4F). For eHIF, the protein was almost completely digested, leaving only minimal fat globules (Figure 4I). Previous research has shown that hydrolyzed milk protein, especially hydrolyzed whey proteins, were more easily digested and broken down into small peptides and amino acids than intact proteins (28). Therefore, the combined actions of pancreatic lipase and bile salt in hydrolyzed infant formula accelerated the speed and increased the extent of protein digestion, resulting in greater hydrolysis of infant formula and making it easier to digest. This result answered the first question raised earlier in the subsection named “Infant formulas with different degrees of hydrolysis were composed of different sized proteins” (Figure 1), namely, why hydrolyzed infant formula was easy to digest.

### Hydrolyzed Infant Formula Could Affect Mouse Growth

**FIGURE 5:**
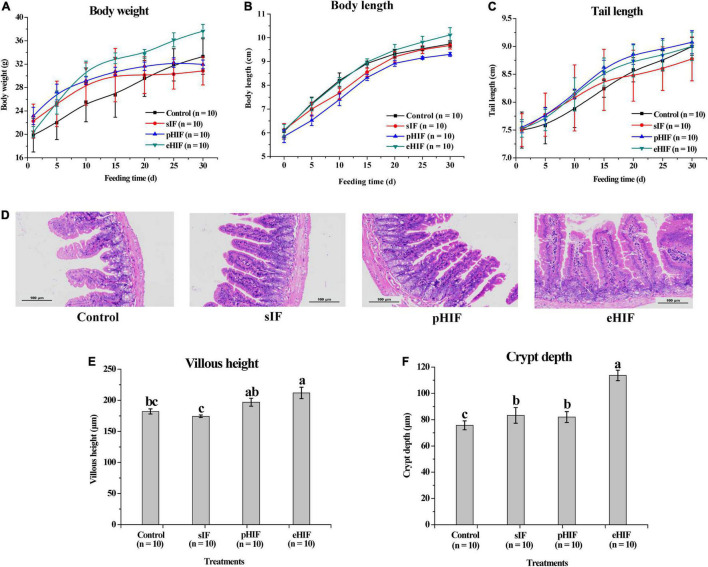
Effects of infant formula on the growth of mice, *n* = 10 independent experiments. Changes in **(A)** body weight, **(B)** body length and **(C)** tail length of mice during the 30-day feeding period. **(D)** Morphology of the small intestines of mice. **(E)** Villus height and **(F)** crypt depth in the small intestines of mice.

Regarding the body weights of mice, each group showed gradual increased during the feeding period (Figure 5A). Compared with the first day of feeding, mice given eHIF had the greatest weight gain (37.66 ± 1.14 g), with a 45.78% increase at the end of the 30-day experiment, followed by the control group (33.30 ± 3.03 g), with a 40.30% increase, and there was no significant difference in the body weight growth rate between them. Mice fed sIF (30.84 ± 2.39 g) and pHIF (31.96 ± 1.27 g) gain the least body weight, 27.82 and 27.53%, respectively, and their body weight gain rate was significantly lower than those of the eHIF and control groups (*p* < 0.05).

With the prolongation of feeding time, the body lengths of mice in each group gradually increased, and the rate of increase in the mice fed pHIF was slower than that in the other three groups (Figure 5B). After 30 days of feeding, the average body length of eHIF group of mice was 10.12 ± 0.30 cm, which was significantly longer than that of the pHIF mice (9.3 ± 0.08 cm; *p* < 0.05).

Regarding tail length, the three groups of mice fed infant formula had faster growing tails than the control mice (Figure 5C). The tail length growth rate in the sIF group slowed down from day 15 and was significantly lower than that in the other three groups by the end of day 30 (*p* < 0.05).

Compared with sIF feeding, pHIF administration had no notable effect on the growth and development of mice, while eHIF had a significant promoting influence. Not only is hydrolyzed infant formula easy to digest, but its small peptide and amino acid degradation products were also easily absorbed (28). Field et al. reported weight gain in mice that received a diet containing hydrolyzed CN for 2 months (44), but they did not measure body length. In this study, the ease of digestibility of eHIF (Figures 1, 4) might have encouraged the mice to absorb more nutrients and assume a larger body size without obvious signs of obesity.

### Hydrolyzed Infant Formula Could Affect Intelligence and Physical Strength in Mice

The maze experiment is a common method used to characterize the spatial learning ability of animals, in which the number of arm entries and turns represent the exploration and spatial memory abilities of experimental animals, respectively (21). There were no significant differences in the number of arm entries or turns between the eHIF and control groups (Table 2). For the sIF group, the number of arm entries was significantly lower than that in the eHIF group (*p* < 0.05), while the number of turns was similar between these two groups. For the pHIF group, the number of arm entries and turns were significantly lower than those of the other three groups (*p* < 0.05). A possible reason for this result was that the abundant fatty acid content in eHIF, especially LCPUFAs (Figure 3), promoted the development of mouse brain (39), thereby improving maze test performance. Fewer LCPUFAs in pHIF (Figure 3) might be responsible for the lower total number of arm entries and turns observed for this group of mice.

**TABLE 2:** Effects of infant formula on intelligence and physical strength in mice.

Test items	Control	sIF	pHIF	eHIF
Number of arm entries	30.3 ± 3.3*^a^*	26.1 ± 2.2*^b^*	17.3 ± 3.6*^c^*	30.1 ± 7.1*^a^*
Number of turns	5.6 ± 0.5*^a^*	5.3 ± 1.4*^a^*	2.6 ± 1.2*^b^*	5.5 ± 2.3*^a^*
Swimming time (s)	195.1 ± 33.0*^a^*	174.0 ± 20.7*^a^*	103.3 ± 42.4*^b^*	86.3 ± 14.0*^c^*

*Means in same row with different small letters are significantly different (p < 0.05). The approve number for animal experiment in each group was 10.*

The mouse weight-bearing swimming test was used to characterize the physical strength of mice in terms of exhaustion time (22), and swimming training could modulate muscle energy metabolism and improve skeletal muscle function (45). There were no significant differences between the control and sIF groups; however, these groups showed significantly longer swimming time than the pHIF group, which displayed a significantly longer time than the eHIF group (*p* < 0.05). The swimming time of the mice in the eHIF group (86.29 ± 14.0 s) was less than half that of the control group (195.1 ± 33.0 s) and the sIF group (174 ± 20.7 s) (Table 2). This indicated that the size of the mice in the eHIF group (Figures 5A,B) did not correspond to their physical strength; instead, these mice appeared to be large in size but weaker. Previous research has shown that lower muscle strength might be associated with underlying muscle disease (46). Therefore, it is necessary to pay attention to the muscle health of infants fed eHIF in the long-term.

### Hydrolyzed Infant Formula Could Affect the Organ Indexes and Intestinal Development in Mice

The ratio of an organ to body weight, also known as the organ index, is an important indicator that can assess the health of an individual organism (23). For the liver index, there was no significant difference between the control and sIF groups, but there was a decreasing trend going from the sIF group to the pHIF and then eHIF groups, and these differences were significant (*p* < 0.05) (Table 3). The liver is an important digestive and metabolic organ that deoxidizes substrates and secretes bile (47). The main nutrients in pHIF and eHIF were hydrolyzed emulsions containing hydrolyzed proteins and small fat globules, so the mice in these groups had a reduced need to secrete bile to digest the formula, which might account for the lower liver indexes in these two groups.

**TABLE 3:** Effects of infant formula on organ index in mice.

Organ index	Control	sIF	pHIF	eHIF
Liver index	1.3 ± 0.4*^a^*	1.3 ± 0.3*^a^*	0.8 ± 0.5*^b^*	0.5 ± 0.1*^c^*
Kidney index	0.4 ± 0.1*^b^*	0.5 ± 0.1*^b^*	1.4 ± 0.5*^a^*	1.4 ± 0.2*^a^*
Spleen index	0.2 ± 0.1*^b^*	0.4 ± 0.1*^a^*	0.3 ± 0.1*^ab^*	0.3 ± 0.1*^ab^*

*Means in same row with different small letters are significantly different (p < 0.05).*

*The approve number for animal experiment in each group was 10.*

For the kidney index, there was no significant difference between the control and sIF groups or between the pHIF and eHIF groups, but the pHIF and eHIF groups had significantly higher kidney index than the control and sIF groups (*p* < 0.05), which was completely opposite to the liver index results (Table 3). The kidneys are metabolic organs that produce urine and excrete metabolic wastes (48). Since the pHIF and eHIF groups were fed only liquid hydrolysate milk, the large amount of water consumed might be the reason for their high kidney indexes.

In terms of spleen index, the sIF group (0.4 ± 0.1) showed a significantly higher value than the control group (0.2 ± 0.1) (*p* < 0.05), pHIF group (0.3 ± 0.1), and eHIF group (0.3 ± 0.1) (Table 3). The spleen is an immune organ, and if there are a large number of macrophages in the spleen, invading antigens and foreign matter can be promptly removed (49). Previous research has demonstrated that formula-fed infants less effectively protect themselves from infection and the active immune system than breast-fed infants (50). Therefore, long-term consumption of infant formula may be detrimental to the development of the immune system in mice, but this hypothesis needs to be confirmed by more experiments in the future.

The digestion and absorption of nutrients in mammals mainly occur in the small intestine. Villus height corresponds to the nutrient absorption area and crypt depth corresponds to intestinal epithelium turnover, so villus height and crypt depth are regarded as indicators of intestinal health and development (51). The integrity, uniformity, and compactness of the intestines of the mice in the pHIF and eHIF groups were significantly better than those in the control and sIF groups (Figure 5D). In addition, the villus height and crypt depth in the eHIF group were significantly better than those in the control, sIF, and pHIF groups (*p* < 0.05) (Figures 5E,F). Higher villi enhanced the surface area of the lumen available for absorption, increased the action of digestive enzymes, and sped up nutrient transport; whereas deeper crypts indicated the rapid turnover of cells, such as absorbing, secreting, and regenerating cells (51). Therefore, eHIF could promote the development of the small intestine in mice.

### Hydrolyzed Infant Formula Could Affect Fecal Microbiota Diversity in Mice

**FIGURE 6:**
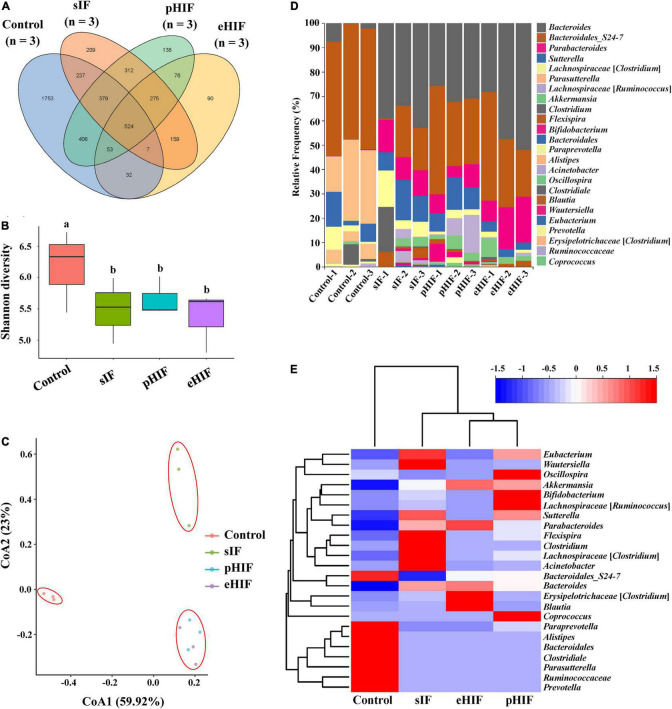
Composition of the fecal microbiota of mice, *n* = 3 independent experiments. **(A)** Venn diagram of ASVs in the fecal microbiota from the mice after the 30-day experiment. **(B)** Cluster analysis of the four treatment groups based on ASVs. **(C)** The correlation of ASVs among the four groups. **(D)** Taxonomic complexity of the bacterial community. **(E)** Heatmap of the abundance of all ASVs.

The mammalian gut microbiota is symbiotic with the host and closely related to host’s immunity, digestion, and metabolism. The composition of the gut microbiota is influenced by the host’s genetic background, foods, and drugs taken in, lifestyle, etc. At present, there are more than 1,000 kinds of gut microbiota in the human body. The short-chain fatty acids produced by these microbiota participate in the emulsification and absorption of lipids from food and affect lipid metabolism and even obesity (52). The diversity of the fecal microbiota can reflect the host’s gut microbiota (53, 54). The fecal microbiota of the four groups of mice were sequenced, and their ASVs were analyzed. There were 1,753 unique ASVs in the control group compared with a total of 1,259 unique ASVs in the other three groups, suggesting that feeding infant formula interfered with the fecal microbiota of mice (Figure 6A). This result is similar to that presented by Yang et al., who suggested that the number of gut microbiota increased when infants switched from cow’s milk to table foods (55). In addition, the control group yielded much higher alpha-diversity values than groups of sIF, pHIF, and eHIF (*p* < 0.05) (Figure 6B), indicating that a lower fecal microbiota richness in infant-fed mice. Moreover, the beta-diversity of the fecal microbiota was also analyzed. The fecal microbiota of mice fed pHIF and eHIF were similar, but they were very different from those of the sIF and control group mice (Figure 6C), suggesting that hydrolyzed infant formula could change the composition of the mouse fecal microbiota.

Formula feeding could also change the composition of fecal microbiota in infants (56, 57). In this study, fecal microbiota composition at the genus level was compared, and it was found that *Bacteroides* were abundant in the feces of mice fed infant formula and were less abundant in the control group, while the results for *Parasutterella* were the opposite, with lower abundance in the feces of formula-fed mice and the lowest in the eHIF group (Figure 6D). A meta-analysis revealed that a lower abundance of *Bacteroides* in the gut microbiota is associated with inflammatory bowel disease (58), suggesting that infant formula could also change the composition of the microbiota to improve healthy intestinal development in mice, especially those fed eHIF (Figures 5D,E,F). The genus *Parasutterella* could alter expression levels of ileal bile acid transporter genes and hepatic bile acid synthesis genes in mice by changing microbial-derived metabolites (e.g., aromatic amino acids, bilirubin, purines, and bile acid derivatives) (59). Healthy adults who consume resistant potato starch to increase levels of *Parasutterella* in the gut microbiome had lower low-density lipoprotein levels (60). Therefore, the lower abundance of *Parasutterella* might be one of the factors for the higher body weights of mice in the eHIF group than in the control group (Figure 5A).

Furthermore, cluster analysis of the 20 most abundant communities showed a significant difference between the three groups fed infant formula and the control group (*p* < 0.05) (Figure 6E). Three groups of infant-fed mice clustered together, showing that feeding infant formula affected their fecal microbiota composition. Similar results have been reported regarding differences in gut microbiota abundance between formula-fed and breast-fed infants (61). Furthermore, our results confirmed that the pHIF mice and eHIF mice were similar, suggesting that hydrolyzed infant formula, and standard infant formula had different effects on the gut microbiota abundance in mice.

## Conclusion

In this study, the hydrolyzed infant formulas of pHIF and eHIF consisted mainly of digestible small-molecular-weight proteins and peptides rather than the large-molecular-weight proteins found in sIF. During the SGF and SIF digestion stages, as the degree of infant formula hydrolysis increased, the molecular weights and allergenicity of the proteins decreased, but the contents of LCPUFAs increased, the digestive solution reached an electrostatic stable state sooner, and the speed of protein and lipid digestion was faster. The eHIF-fed mice were larger but weaker. Although eHIF promoted spatial learning ability and small intestine development in mice and changed their gut microbial composition, eHIF might be detrimental to their immune system.

## Data Availability Statement

The datasets presented in this study can be found in online repositories. The names of the repository/repositories and accession number(s) can be found in the article/Supplementary Material.

## Ethics Statement

The animal study was reviewed and approved by School Animal Care and Ethics Committee of Zhejiang Gongshang University.

## Author Contributions

LF and JH conceived, designed research, and wrote the manuscript. LF, WY, KZ, and MW conducted the experiments. LF, DQ, and WL analyzed the data. All authors have read and agreed to the published version of the manuscript.

## Conflict of Interest

The authors declare that the research was conducted in the absence of any commercial or financial relationships that could be construed as a potential conflict of interest.

## Publisher’s Note

All claims expressed in this article are solely those of the authors and do not necessarily represent those of their affiliated organizations, or those of the publisher, the editors and the reviewers. Any product that may be evaluated in this article, or claim that may be made by its manufacturer, is not guaranteed or endorsed by the publisher.

## Supplementary Material

Supplementary Table 1 Infant formula nutrition facts per 5 FL OZ.
